# Force plate coverings significantly affect measurement of ground reaction forces

**DOI:** 10.1371/journal.pone.0293959

**Published:** 2023-11-03

**Authors:** Tina Smith, Massimiliano Ditroilo

**Affiliations:** 1 Faculty of Education, Health & Wellbeing, University of Wolverhampton, Walsall, United Kingdom; 2 School of Public Health, Physiotherapy and Sports Science, University College Dublin, Dublin, Ireland; Kennedy Krieger Institute/Johns Hopkins University School of Medicine, UNITED STATES

## Abstract

The purpose of this study was to carry out a material test to investigate the effect of different force plate coverings on vertical and horizontal ground reaction force and derived parameters. Four surface conditions were analysed; bare plate, vinyl, sportflex, and astroturf on a Kistler force plate. Vertical data were collected by dropping a 2 kg rigid, textured medicine ball from a low (61 cm) and a high (139 cm) height. Horizontal data were collected using a custom-built, rigid, metal pendulum device. A one-way ANOVA revealed a significant main effect of surface on peak force and rate of force development for high height, low height, and horizontal force conditions (all p<0.001), with effect sizes in the post-hoc analysis being mostly large to very large. Interestingly, sportflex yielded the highest vertical but the lowest horizontal ground reaction forces. This study showed the use of current force platform coverings had a significant effect on peak force and rate of force development measurements during a standardised testing procedure. Future research should try to obtain rate of force development values that more closely replicate aspects of human performance during standardised testing procedures. Also further investigate the effect of the different surfaces on ground reaction forces during human movement.

## Introduction

Biomechanics laboratories designed for researching the kinetics of sporting movements often incorporate force plates embedded in the floor to measure ground reaction forces (GRF).

One common practice is to place a covering on the laboratory floor and over the force plates to replicate the surface used in sporting competition, increasing ecological validity of analysis. Further, force plate coverings disguise the force plate from participants, reducing the likelihood of participants artificially altering the movement being measured.

However, little is known about how much a covering surface alters GRF and derived parameters in these specific laboratory-based circumstances. Early work clarified methodological aspects of how to assess sports surfaces using material tests and tests involving human participants, in order to gain an insight into surface-related injuries and performance enhancement [1–3]. Material tests typically involve dropping a mass, in standardised conditions, onto the surface under investigation, whereas in tests involving participants an individual performs relevant movements on the surface of interest [2].

When different surfaces were compared using a material test, parameters such as peak deceleration and rate of deceleration of the mass dropped onto the surface were significantly different between surfaces [4]. However, when participants performed heel-toe running trials similar peak impact forces were found for the different surfaces; which were explained by individual kinematic adjustments to running motion [4]. A similar outcome was observed in the study by Ferris et al [5]. The authors found that when runners had to step onto surfaces of different stiffness, they adjusted leg stiffness to ensure a stable transition between surfaces without changing the path of their centre of mass.

However, the association between kinematic adjustments and maintaining impact forces across different surfaces is not as clear in non-repetitive tasks, such as a running tennis forehand foot plant, where kinematics adjustments have been observed as less consistent despite similar impact forces [6].

Even though the individual kinematic adjustments to different surfaces is a research area with interesting applications [7], further material testing is also warranted. Literature on material tests relevant to assessment within biomechanical laboratories is scant, and choice of surfaces in the aforementioned studies is dated. Having a greater insight into the mechanical effect of contemporary surface materials may help the interpretation of participant generated kinetic data and any associated kinematic adjustments, when collected from different environments/laboratories and using different force plate covering.

The aim of this study was to investigate differences in vertical and horizontal GRF and derived parameters (Rate of force development, RFD) between surface types, using contemporary surfaces and standardised tests. To make the experiment relevant to sporting movements, we have endeavoured to generate GRF data that replicate, as closely as possible, those expected in this type of activity. We hypothesised that, due to differences in material properties and thickness, GRF and RFD will differ between the four surfaces tested.

## Methods

Two types of tests were performed to assess vertical and horizontal force under four conditions of platform surface covering. The four surface conditions were; bare plate, no covering (Bare); vinyl floor covering (Vinyl); Sportflex athletics track surface (Sportflex) (Mondo, Rugby, UK); and a multi-sport astroturf covering with no infill (Astroturf) (As Good As Grass, Preston, UK). Surfaces were cut to be within the dimensions of the force platform and adhered to the platform using double-sided tape.

*Vertical data* were collected by dropping a 2 kg rigid, textured medicine ball onto a Kistler 9286AA force platform (Kistler, Winterthur, Switzerland; sampling frequency 5000Hz) from two known heights (low: 61 cm; high: 139 cm). The object and heights were chosen to replicate the magnitude of GRF generated during human gait [8]. The medicine ball was held at the specified height against a fixed structure and manually released. A total of 60 trials were repeated for each height and surface condition.

**Fig 1:**
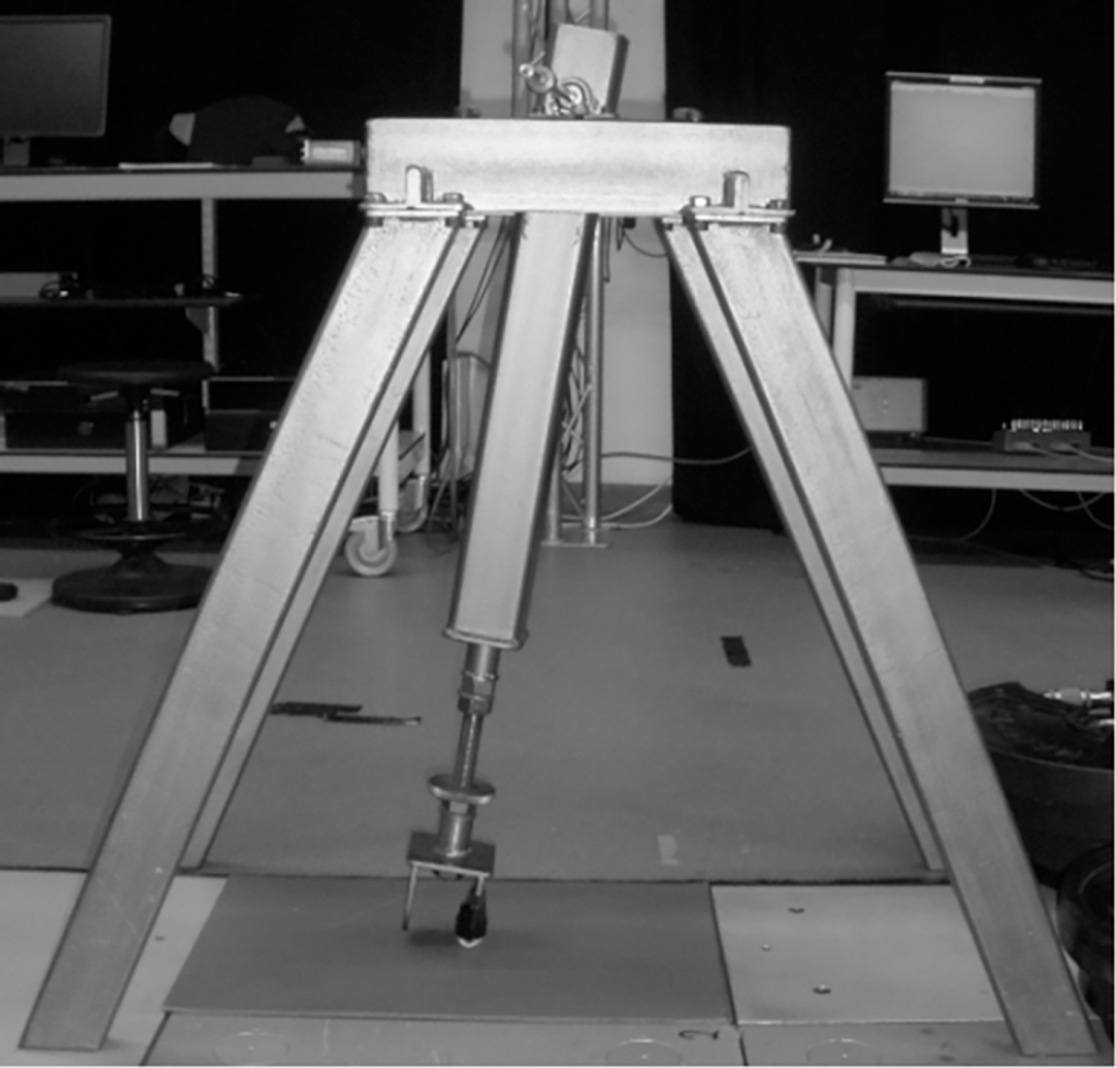
Custom-built pendulum device used to collect horizontal ground reaction force data. The contact point of the pendulum arm was composed of solid metal with a protective covering constructed from the outer shell of a standard tennis ball. The pendulum arm was retracted to a standardised angle each trial and manually released by the same researcher, before making contact with the force platform.

*Horizontal data* were collected using a custom built, rigid, metal pendulum device (Fig 1).

The pendulum arm was retracted to a standardised angle each trial and manually released by the same researcher. The arm made contact with a Kistler 9281B force platform (Kistler, Winterthur, Switzerland; sampling frequency 5000Hz). The contact point of the pendulum arm was composed of solid metal with a protective covering constructed from the outer shell of a standard tennis ball. A total of 60 trials were completed for each surface.

**Fig 2:**
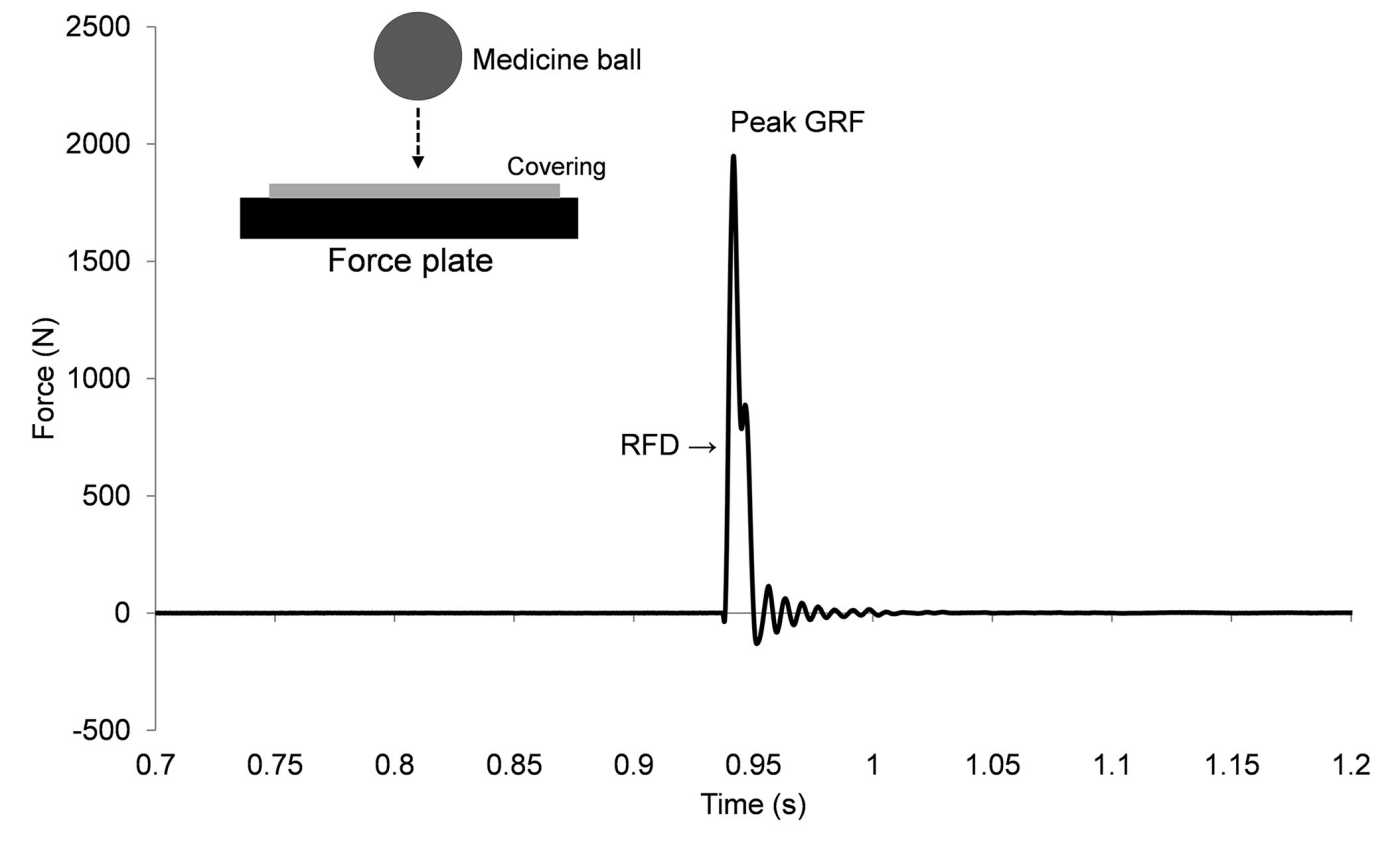
Raw force/time trace following a ball drop. GRF = ground reaction force; RFD = rate of force development.

For both vertical and horizontal data, the two lowest and two highest values were removed and the remaining 56 trials were used for later analysis. The raw GRF data (Fig 2) were used to determine: peak force (PF, N), as the greatest force value recorded when the object was in contact with the force plate; and RFD (N/s), which was calculated as the slope of the GRF from onset to peak as follows:

$$RFD=\frac{PF-OF}{t_{PF}-t_{OF}}$$

where onset of force (OF), is the point at which the GRF exceeded the baseline level by 10 N, and t_PF_ and t_OF_ are the times at which PF and OF occurred, respectively.

For each surface, a total of six variables were identified: three PFs and three RFDs, representing vertical GRF for low and high conditions, and horizontal GRF.

Statistical analysis was carried out using Jamovi (version 2.3, The jamovi project, jamovi.org). Normality of distribution was confirmed using a Kolmogorov-Smirnov test. A one-way ANOVA was applied to each of the six variables, comparing the four conditions. When a significant difference was found, a Tukey HSD post-hoc analysis was used to identify where the differences lay, and Cohen’s d effect size (ES) was calculated. Statistical significance was set at p < 0.05 and ES was interpreted as trivial (0.00–0.19), small (0.20–0.59), moderate (0.60–1.19), large (1.20–1.99) and very large (≥ 2.00) [9].

## Results

**Fig 3:**
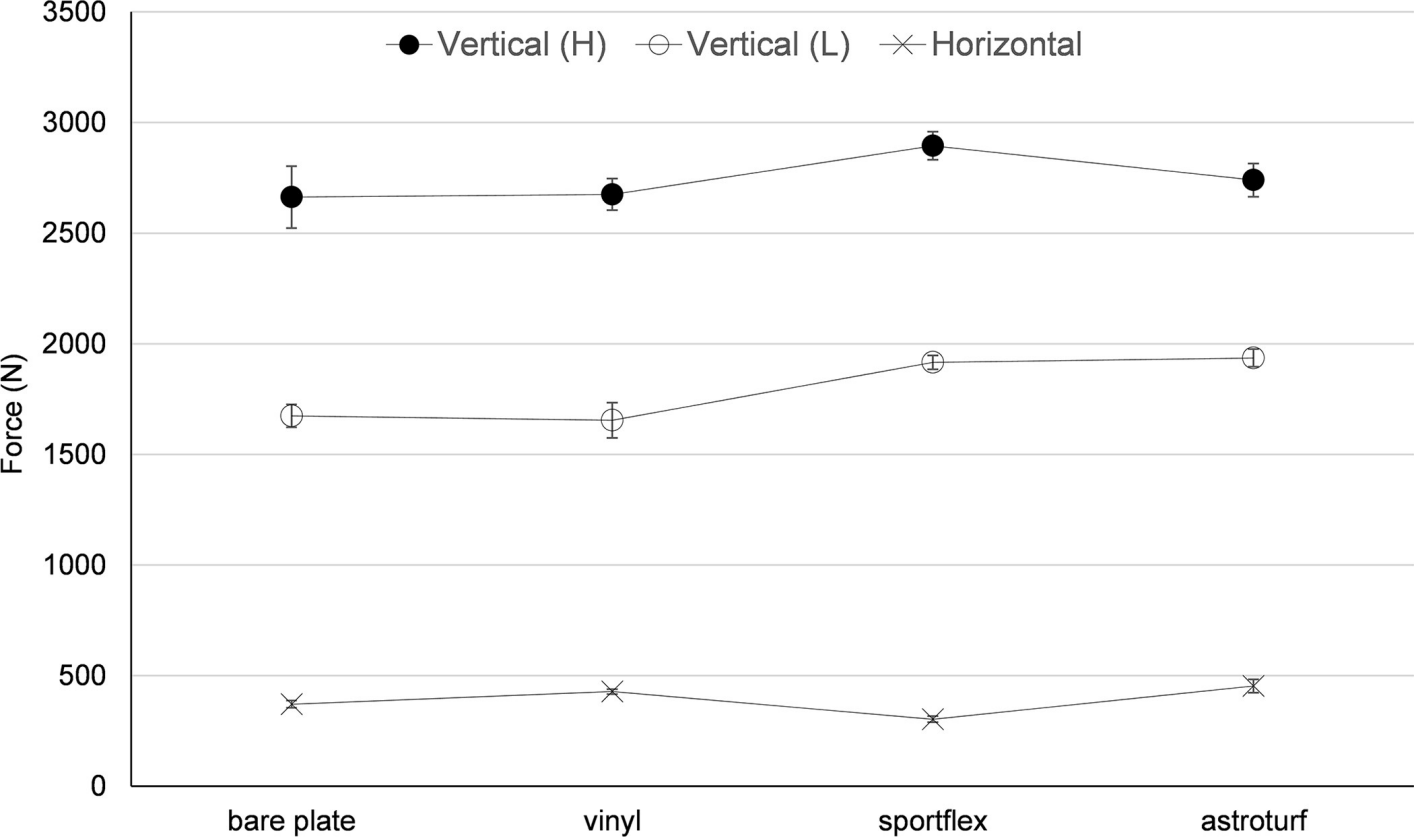
Vertical and horizontal ground reaction forces (mean and SD) recorded under different conditions. H = drop from high height; L = drop from low height.

**Fig 4:**
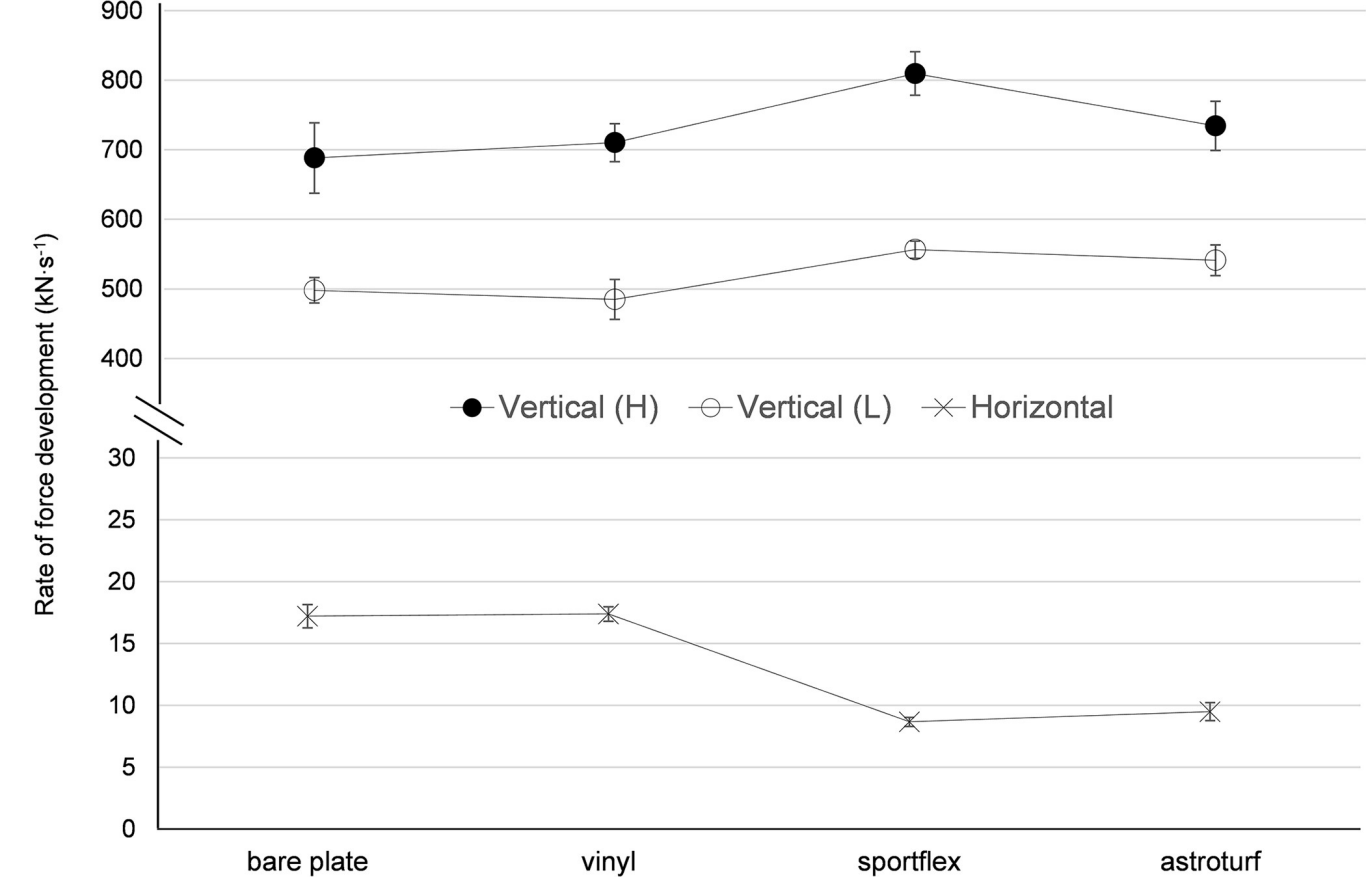
Rate of force development (mean and SD) calculated from ground reaction force curves recorded under different conditions. H = drop from high height; L = drop from low height.

Figs 3 and 4 show the mean values (±SD) for peak force and RFD recorded in the three conditions with the four plate coverings, respectively. A significant main effect for surface was observed for peak force (Fig 3), high height (F = 422, p < 0.001), low height (F = 2759, p < 0.001) and horizontal force (F = 2910, p < 0.001). Post-hoc tests revealed that there were significant differences between all surfaces (p < 0.01) for low and high height vertical force, and horizontal force conditions, except for bare plate vs vinyl (high height, p = 0.33, ES = 0.12). ES was small (low height, Bare vs Vinyl, ES = 0.31; and Sportflex vs Astroturf, ES = 0.55), moderate (high height, Bare vs Astroturf, ES = 0.71; and Vinyl vs Astroturf, ES = 0.87) and large to very large for all other comparisons (ES 1.22 to 6.74).

Similarly, a significant main effect for surface was observed for RFD (Fig 4) for high height (F = 1347, p < 0.001), low height (F = 1236, p < 0.001) and horizontal force (F = 15867, p < 0.001). Post-hoc tests revealed that there were differences between all surfaces (p < 0.01) for low and high height vertical force, and horizontal force conditions. ES was small (horizontal, low and high height, Bare vs Vinyl, ES = 0.24, 0.56, 0.57, respectively), moderate (high height, Bare vs Astroturf, ES = 1.08; low height, Sportflex vs Astroturf, ES = 0.88) and large to very large for all other comparisons (ES 2.15 to 18.33).

## Discussion

As little is known about the effect of force platform surface coverings on GRF, this study aimed to investigate differences in vertical and horizontal GRF and derived parameters between surface type, using contemporary surfaces and standardised tests. The results indicate that attaching a surface on top of a force platform significantly influences the vertical and horizontal peak forces and RFDs recorded by the platform in a standardised simulation study, thus we accept our hypothesis. In most cases there were large to very large effect sizes found for comparisons between all surfaces.

The above findings have potential implications for research and clinical practice when comparing results to existing datasets that used force platforms with different coverings, as GRF values for the same movement may differ due to surface type. In addition, differences will also be reflected in any subsequent calculations made, such as calculation of joint moments.

Although some studies have indicated performers make kinematic adjustments relevant to the surfaces they make contact with in order to maintain similar GRFs [4, 5], the types of adjustments to motion may not be consistent [6]. It is possible that performing an open skill, where the environment dictates the nature of the movement (e.g. tennis ground stroke) may distract participants from consciously adjusting to the surface [10]. Similarly, we do not fully understand if competence or ability at a specific task affects capacity to adjust to surface, as the degree of focus on the task at hand may affect this [10]. However, the above suggestions do not seem relevant to running where it appears more adjustments in relation to different surfaces occur, resulting in more consistent external loading variables across surfaces [11]. Past research is however limited with respect to type of movement and populations studied when investigating the effect of surface on GRF; therefore the extent to which task and ability affects participants consciously adjusting to the surface is not clear and warrants further investigation. As we have demonstrated, surface type affects GRF under standardised conditions, and uncertainties around surface type affecting GRF data during human movement remain. We recommended future studies report force plate surface covering details, allowing for researchers to make objective judgements when comparing findings between studies.

The current study results also demonstrated a notable exception to the large and very large effect sizes for comparisons between the bare plate and when covered with a vinyl surface. For this comparison only the peak horizontal force showed a very large effect size between surfaces, with all others being small or non-significant. Surface thickness has been proposed as more important in altering peak force than the type of material itself [12]. In the current study, the thickness of the surface varied from 5 mm (vinyl) to 13.5 mm (Sportflex and astroturf). The small differences in thickness between bare plate and vinyl conditions could be a contributing factor that had less influence when considering horizontal force. Additionally, the non-sport vinyl surface may not have been designed with reducing impact force as a consideration. However, despite similar thickness, small (0.55) to very large (6.74) effects were seen for the peak force and RFD comparisons between Sportflex and Astroturf, indicating the material properties of the differing types of surface also had significant implications. For example the Sportflex surface used in the current study was designed to minimise foot contact time and maximise energy return to optimise running performance, whereas the astroturf surface tested was not [13].

As different materials are likely to respond differently to different types of applied forces [2], two ranges of vertical force application were used in this study. For both high and low height vertical test scenarios, the results demonstrated a relatively consistent pattern in responses (Fig 3). However, the pattern was different for horizontal force testing; where the peak force and RFD recorded for the Sportflex material were the lowest values of all surfaces despite being the highest values for both vertical testing conditions (Fig 3). It is possible that the unique construction of the Sportflex surface meant it was the only tested material that was designed to deform in multiple dimensions [13]; highlighting the relative importance of considering surface construction and whether inherent design features affecting specific GRF parameters are present. It is possible that the Sportflex surface, which is designed for athletics, may not have been optimal for sprint performance, as horizontal force has been indicated as a major determinant of 100m performance [14].

A limitation of this study is that the RFD values obtained (Fig 4) are larger than those recorded during some human movements, such as walking and running [15, 16]. However, Stiles and Dixon [6] report values of loading rate during a running tennis forehand foot plant very close to those of the current study (vertical drop, low height). Shortcomings of previous surface testing studies include their use of materials or applied forces that are not representative of sporting movements [2]. Therefore, the surfaces used in the current study were contemporary, as used in athletics and multi-sport activities. Further, the test method and materials used in the current study were such that peak force values were close to those previously reported for human movement (e.g. low height < 2000 N; high height < 3000 N; horizontal < 500 N). However, this inexpensive and ecologically valid approach of standardised testing could be improved in order to better replicate RFDs within the typical human range. In this regard, future work should consider using a softer object to drop on the force plate. As a result, a reduced slope of the load/deformation curve would occur, which in turn would lessen RFD values closer to those typically seen within the human range [17].

## Conclusion

This study showed that the use of different force platform coverings has a significant effect on peak force and rate of force development measurements during standardised testing aimed at replicating specific kinetic aspects of human performance Researchers should therefore consider the effect of force platform coverings with respect to the likely loads applied during the test scenario and their potential to affect the experiment before using surface covered force platforms.

To explore the effects of different surfaces on contact forces, future studies should consider participant-based testing alongside standardised testing that generates force parameters of similar magnitude to the activity of interest. It is recommended that these studies also incorporate investigating the potential effect of different tasks and participant ability on the degree of kinematic adjustments made in relation to the surface.
